# Developmental potential of non- and mono-pronuclear zygotes and associated clinical outcomes in IVF cycles

**DOI:** 10.3389/fendo.2024.1361734

**Published:** 2024-03-12

**Authors:** Mengyi Zhu, Qiyin Dong, Yurong Zhu, Yun Le, Tengfei Wang, Yuanping Zhou, Sheng Yang

**Affiliations:** Department of Assisted Reproduction, Huzhou Maternity & Child Health Care Hospital, Huzhou, Zhejiang, China

**Keywords:** 0PN and 1PN zygotes, blastocyst culture, developmental potential, pregnancy rates, neonatal outcomes

## Abstract

**Purpose:**

This study aims to evaluate the developmental potential of 0PN, 1PN, and 2PN zygotes in IVF cycles and compare their clinical outcomes.

**Methods:**

We conducted a retrospective cohort study involving IVF patients. Blastocyst formation rates were assessed with 0PN, 1PN, and 2PN zygotes. Subsequently, we collected clinical outcome data following the transfer of these zygotes.

**Results:**

The overall blastulation rate was similar between 0PN (29.6%) and 2PN (32.1%) zygotes, but 1PN zygotes exhibited a significantly lower blastulation rate (17.0%) compared to both 0PN and 2PN zygotes. Similarly, the overall rate of good-quality blastulation was comparable between 0PN (15.3%) and 2PN (17.5%) zygotes, while 1PN zygotes showed a significantly lower rate (7.0%) compared to both 0PN and 2PN. Clinical pregnancy, ectopic pregnancy, implantation, and live birth rates were similar among single blastocyst frozen embryo transfers (FET) of 0PN, 1PN, and 2PN. Additionally, no significant differences were observed between single- and double-blastocyst FET of 0PN and 2PN.

**Conclusions:**

Our findings suggest that 0PN and 2PN zygotes have comparable developmental potential, while 1PN embryos exhibit lower developmental potential. Blastocyst FET outcomes appear similar among 0PN, 1PN, and 2PN zygotes.

## Introduction

In the field of assisted reproduction, successful fertilization is determined by the observation of zygotes exhibiting two distinct pronuclei (2PN) and two polar bodies around 16–18 h after insemination. However, 0PN and 1PN zygotes, lacking pronuclei or featuring a single pronucleus, are not uncommon during *in vitro* fertilization (IVF) cycles, with reported incidences ranging from 11% to 20% for 0PN and 4% to 8% for 1PN (1–5). Generally, embryos derived from 0PN or 1PN zygotes are considered failed or abnormal fertilization events and are not the most suitable ones for current clinical implementation (6). Due to the potential risk of chromosomal abnormalities, these embryos are usually not preferred at first transplantation. However, in some cases, there may be an insufficient number of good-quality 2PN embryos available for transfer. It is worth noting that certain 0PN- and 1PN-derived embryos are diploid and possess a normal chromosomal structure (7, 8). These embryos have the potential to develop into high-quality blastocysts similar to those derived from normally fertilized 2PN embryos (7–10). Additionally, it is important to recognize that haploidy can also occur in 2PN-derived embryos (11). For patients who do not have 2PN embryos available for transfer, the option of using 0PN- or 1PN-derived blastocysts could be considered (9, 12–14).

Preimplantation genetic testing (PGT) is a highly effective method for screening diploid embryos. However, not all reproductive centers are qualified to perform PGT. While PGT carries some risks as an invasive procedure (15), blastocyst culture remains an indispensable noninvasive, convenient, and cost-effective method in clinical practice. This is because a significant number of haploid embryos fail to develop to the blastocyst stage, resulting in a higher diploid rate among blastocysts compared to cleavage-stage embryos (16). Previous studies have shown that the transfer of blastocyst embryos derived from 0PN and 1PN can actually improve the chances of a successful pregnancy. In contrast, the clinical outcomes of cleavage-stage 0PN and 1PN embryos were significantly lower compared to those of 2PN embryos (11, 13). The quality and implantation potential of blastocysts can be predicted through morphological assessments of cleavage-stage embryos, including factors such as blastomere number, cellular fragmentation, and evenness of cell size and symmetry (17). Furthermore, only a few retrospective studies have reported on the potential health risks associated with 0PN and 1PN embryo offspring (12, 18). Therefore, further investigation is needed to ensure the safety of 0PN and 1PN embryo transfers.

A retrospective study was conducted to assess the clinical utility of embryos derived from 0PN and 1PN fertilization. The study examined pregnancy outcomes, neonatal health, and the incidence of abortion following the transfer of blastocysts derived from both types of embryos. The results of this study offer valuable insights into the clinical significance of 0PN and 1PN embryos, as well as the well-being of offspring resulting from their utilization.

## Materials and methods

### Patients and cycles

In this retrospective study, the outcomes of IVF embryos were classified into three groups: 0PN, 1PN, and 2PN. The study time frame was oocyte collections from January 2020 to December 2021, whereas clinical outcomes included frozen-thawed embryo transfer cycles using the results from the same time period. Our study included patients aged 20 to 45 years. The exclusion criteria were as follows: intracytoplasmic sperm injection cycles and loss to follow-up. This study was conducted with the approval of the ethics committee of Huzhou Maternity and Child Health Care Hospital.

### Stimulation protocols

The ovarian stimulation protocols were chosen depending on each patient’s age, whether older than 35, serum anti-Müllerian hormone (AMH) level, and body mass index (BMI). Women underwent controlled ovarian stimulation using a standard. Medication was then administered with recombinant follicle-stimulating hormone (rFSH), GONAL-F (Merck Serono, Europe), in which younger than 35-year-old patients were advised to take two ampoules (150 IU) of rFSH daily, and older than 35-year-old patients were advised to take three or four ampoules (225 IU or 300 IU) of rFSH daily.

### Laboratory protocols

Approximately 34–36 h after the human chorionic gonadotrophin (hCG) trigger, the oocytes were aspirated and fertilized with conventional insemination. Spermatozoa were harvested with the use of density gradient centrifugation and swim-up methods with 100,000 motile sperm/mL in the insemination dish. Embryos were cultured in the microdroplet medium covered with mineral oil (Vitrolife, Sweden) in culture dishes. In detail, 4 h after follicle retrieval, the collected cumulus–oocyte complex were inseminated and cultured in G-IVF PLUS medium drop (50 µL) (Vitrolife, Sweden) at 37°C in a humidified atmosphere of 6% CO_2_ and 5% O_2_ (Thermo Fisher Scientific, USA). When two pronuclei were seen 16–18 h after insemination, the fertilization was regarded as normal. Abnormal zygotes with no sign of pronuclei were divided as 0PN. The number of pronuclei observed was recorded as one, which was also abnormal. Zygotes with 2PN as well as 0PN and 1PN were further cultured. Zygotes were transferred to G-1™ PLUS medium drop (30 µL) (Vitrolife, Sweden) individually after fertilization assessment. According to the embryo grade on day 3, the embryos with the same grade were cocultured in a G-2™ PLUS medium drop (30 µL) (Vitrolife, Sweden) until the blastocyst stage was reached. However, there are no more than five embryos per drop. The embryos were transferred to fresh G-2™ PLUS medium drops and further cultured to day 6 post-IVF. G-1™ PLUS and G-2™ PLUS were placed in dry incubators (37°C, 6% CO_2_, and 5% O_2_) (Astec, Japan). Observations were made based on the morphology of the day 3 (67 h post-insemination) (19, 20) embryos and evaluated by the following scoring system: grade I—the cell numbers ranged between 6 and 12, the embryos developed at a normal rate, the blastomeres were uniform, the cytoplasm was clear, there were no vacuoles, and there was only 5% embryo fragmentation; grade II—the cell numbers ranged between 6 and 12, the blastomeres were slightly uneven uniform, the morphology was slightly irregular, the cytoplasm was clear, there were no vacuoles, and the fragmentation was between 5% and 20%; grade III—the cell numbers ranged between 6 and 12, the embryo development was roughly normal, the blastomeres were uneven uniform, there were few vacuoles in the cytoplasm, and the fragmentation was between 20% and 50%; and grade IV—the number of cells was ≤ 5 or ≥ 13, the embryo development rate was abnormal, the blastomeres were severely unequally sized, there were significant cytoplasmic particles, there was a high quantity of vacuoles, and the fragmentation was >50%. The blastocyst stage was scored according to Gardner and Schoolcraft’s classification (21). Based on these criteria, we defined good-quality blastocysts as expansion grade ≥ 3, inner cell mass grade ≥ B, and trophectoderm grade ≥ B on day 5 (115 h post-insemination) and day 6 (139 h post-insemination) (19, 20). Blastocysts that contained one C on days 5 and 6 were classified as usable embryos.

### Embryo transfer

The endometrium was prepared by a modified natural cycle, a stimulated cycle, or an artificial cycle according to individual differences. In our present study, only one or two usable blastocysts were transferred in every transfer cycle. After embryo transfers, luteal support was initiated. When transplanting embryos, good-quality blastocysts derived from 2PN embryos were given priority, but in cases where no other blastocysts were available within 2PN, the 0PN and 1PN embryos were chosen for transfer.

### Follow-up for pregnancy rates

The pregnancy rates of all patients in the study were followed up until birth. All infants delivered were evaluated for any signs of complications. The patient’s serum hCG concentration was measured on day 14 after blastocyst transfer. A positive hCG test indicates a biochemical pregnancy. Clinical pregnancy was confirmed by the observation of a pregnancy sac with an ultrasound examination on day 35 after embryo transfer. Pregnant women who suffered a miscarriage before the 22nd gestational week were defined as having had an intrauterine abortion. Live birth was defined as the delivery of a living infant at ≥ 22nd weeks of gestation (22).

### Statistical analysis

Data analysis was performed using SPSS software. The data were assessed for normality of distribution by the Kolmogorov–Smirnov (*N* > 50) or Shapiro–Wilk (*N* ≤ 50) tests. Those normally distributed data were presented as the standard deviation and analyzed using Student’s *t*-test. Those significantly skewed were presented as mean ± standard and were performed using the Mann–Whitney *U*-test. Data for multiple rates were analyzed using the Chi-square test. A *p*-value less than 0.05 was considered statistically significant.

## Results

In this study, a total of 18,734 oocytes from 1,628 IVF cycles and 1,311 patients were reviewed. 10,642 embryos were cultured to the blastocyst stage, comprising 705 0PN, 412 1PN, and 9,525 2PN embryos. Blastocyst development was assessed in these embryos, revealing blastocyst rates of 29.6% (209/705) for 0PN, 17.0% (70/412) for 1PN, and 32.1% (3,058/9,525) for 2PN. Additionally, good-quality blastocyst rates were 15.3% (108/705) for 0PN, 7.0% (29/412) for 1PN, and 17.5% (1,663/9,525) for 2PN. Our data revealed that 1PN embryos have a low developmental potential compared with 0PN and 2PN embryos. Significantly less 1PN develop into blastocysts and good-quality blastocysts compared to 2PN (*p* < 0.001; **Tables 1**, **2**). Both blastocyst and good-quality blastocyst rates were significantly higher in the 0PN group when compared with the 1PN group (*p* < 0.001; **Tables 1**, **2**). There was no difference between the 0PN and the 2PN groups. Further studies have found that the result of blastulation culture in women less than 35 years was similar to those without age limits (*p* < 0.001; **Tables 1**–**3**). However, there was no statistical difference in blastocyst and good-quality blastocyst rates in women over 35 years (**Table 3**).

**Table 1:** Total blastulation rate of abnormally fertilized (0PN and 1PN) and normally fertilized (2PN).

Day 3 embryo quality	0PN	1PN	2PN	*P*1	*P*2	*P*3
I–II	35.8% (123/344)	28.3% (47/166)	56.3% (1,406/2,498)	NS	< 0.001	< 0.001
III	32.6% (60/184)	13.4% (16/119)	40.2% (1,073/2,670)	< 0.001	< 0.05	< 0.001
IV	14.7% (26/177)	5.5% (7/127)	13.3% (579/4357)	< 0.05	NS	< 0.05
Total	29.6% (209/705)	17.0% (70/412)	32.1% (3,058/9,525)	< 0.001	NS	< 0.001

P1, p-value between 0PN and 1PN; P2, p-value between 0PN and 2PN; P3, p-value between 1PN and 2PN; NS, nonsignificant.

**Table 2:** Good-quality blastulation rate of abnormally fertilized (0PN and 1PN) and normally fertilized (2PN).

Day 3 embryo quality	0PN	1PN	2PN	*P*1	*P*2	*P*3
I–II	21.2% (73/344)	13.9% (23/166)	33.7% (843/2,498)	< 0.05	< 0.001	< 0.001
III	14.1% (26/184)	4.2% (5/119)	22.0% (587/2670)	< 0.05	< 0.05	< 0.001
IV	5.1% (9/177)	0.8% (1/127)	5.3% (233/4,357)	NS	NS	< 0.05
Total	15.3% (108/705)	7.0% (29/412)	17.5% (1,663/9,525)	< 0.001	NS	< 0.001

P1, p-value between 0PN and 1PN; P2, p-value between 0PN and 2PN; P3, p-value between 1PN and 2PN; NS, nonsignificant.

**Table 3:** Age factors associated with blastulation rate and good-quality blastocyst rate.

	0PN	1PN	2PN	*P*1	*P*2	*P*3
Female age ≤35						
Blastulation rate	29.4% (184/625)	16.9% (60/354)	32.8% (2,772/8,449)	< 0.001	NS	< 0.001
Good-quality blastulation rate	15.2% (95/625)	6.8% (24/354)	18.0% (1,525/8,449)	< 0.001	NS	< 0.001
Female age >35						
Blastulation rate	31.3% (25/80)	17.2% (10/58)	26.6% (286/1,076)	NS	NS	NS
Good-quality blastulation rate	16.3% (13/80)	8.6% (5/58)	12.8% (138/1076)	NS	NS	NS

P1, p-value between 0PN and 1PN; P2, p-value between 0PN and 2PN; P3, p-value between 1PN and 2PN; NS, nonsignificant.

Subsequently, the 0PN, 1PN, and 2PN embryos were subdivided into three groups (I and II, III, and IV) based on the different grades of the day 3 cleavage embryos to further analyze the blastocysts rate. We evaluated the cleavage stage embryos at 67 h post-insemination, which was consistent with the reported embryos assessment time on day 3 (19, 20). Both blastocyst and good-quality blastocyst rates were significantly higher in the 2PN group when compared to the 1PN group, regardless of the embryo quality assessed on day 3 (*p* < 0.05; **Tables 1**, **2**). Further comparison revealed that in 2PN, grades I–III embryos presented higher blastocyst and good-quality blastocyst rate when compared with the same level in 0PN zygotes (*p* < 0.05; **Tables 1**, **2**). We also found that 0PN III and IV embryo blastocyst rates were significantly higher than 1PN (*p* < 0.05; **Table 1**). However, 0PN I–III embryos’ good-quality blastocyst rates were significantly higher than 1PN (*p* < 0.05; **Table 2**).

We then evaluated the blastocyst and the good-quality blastocyst rates on 0PN, 1PN, and 2PN formation on day 5 (115 h post-insemination) and day 6 (139 h post-insemination) (19, 20). For example, 9.7% of 1PN embryos from IVF were capable of reaching the blastocyst stage by day 5, compared with 20.8% of 2PN embryos (*p* < 0.001, **Table 4**), and significantly fewer 1PN-derived blastocysts were of good quality (5.1% and 13.6%; *p* < 0.001, **Table 4**), respectively. Moreover, 0PN embryos generated by IVF had a higher developmental potential compared with 1PN embryos; for example, 21.8% and 9.7% reached the blastocyst stage by day 5, 12.9% and 5.1% reached the good-quality blastocyst stage (*p* < 0.001, **Table 4**), respectively. On day 6, blastocyst and good-quality blastocyst formation rate were higher in 2PN, whether compared to 0PN or 1PN (*p* < 0.05; **Table 4**).

**Table 4:** Blastulation rate and good-quality blastocyst rate of 0PN, 1PN, and 2PN on day 5 or 6.

	0PN		1PN		2PN		*P*1	*P*2	*P*3
	Day 5	Day 6	Day 5	Day 6	Day 5	Day 6	*P*4	*P*5	*P*6
Blastulation rate	21.8% (154/705)	7.8% (55/705)	9.7% (40/412)	7.3% (30/412)	20.8% (1,981/9,525)	11.3% (1,077/9,525)	< 0.001	NS	< 0.001
							NS	< 0.05	< 0.05
Good-quality blastulation rate	12.9% (91/705)	2.4% (17/705)	5.1% (21/412)	1.9% (8/412)	13.6% (1,291/9,525)	3.9% (372/9,525)	< 0.001	NS	< 0.001
							NS	< 0.05	< 0.05

P1, p-value between 0PN and 1PN on day 5; P2, p-value between 0PN and 2PN on day 5; P3, p-value between 1PN and 2PN on day 5; P4, p-value between 0PN and 1PN on day 6; P5, p-value between 0PN and 2PN on day 6; P6, p-value between 1PN and 2PN on day 6; NS, nonsignificant.

A total of 35 single 0PN blastocyst transfers, 18 single 1PN blastocyst transfers, and 153 single 2PN blastocyst transfers were conducted. There were no significant differences in biochemical pregnancy, clinical pregnancy, ectopic pregnancy, implantation, miscarriage, delivery, or live birth rates among these groups. Significantly fewer times of cumulative transfer per woman, including both fresh transfer and frozen embryo transfer cycles, were in the 2PN group compared to the 0PN (*p* < 0.05; **Table 5**). In the group, ten 0PN singletons, seven 1PN singletons, and sixty 2PN singletons neonatal babies were delivered. The birth weight and birth height were also similar among the three groups. We next examined the chromosome content of miscarriage derived from 0PN and 1PN embryos. For the five cases, trisomy was observed in one miscarriage (one of five); the remaining four were normal. So far, no neonatal abnormalities have been reported.

**Table 5:** Comparison of single blastocyst transferred cycles resulting in clinical outcomes originating from 0PN, 1PN, and 2PN.

	0PN	1PN	2PN	*P*1	*P*2	*P*3
Cycle (*n*)	35	18	153	–	–	–
Age (year)	32.3 ± 5.8	32.0 ± 6.1	30.1 ± 4.3	NS	NS	NS
Times of cumulative transfer per female (*n*)	2.0 ± 0.8	2.0 ± 1.2	1.7 ± 0.9	NS	< 0.05^a^	NS
Biochemical pregnancy rate (%)	48.6 (17/35)	55.6 (10/18)	55.6 (85/153)	NS	NS	NS
Clinical pregnancy rate (%)	40.0 (14/35)	44.4 (8/18)	49.7 (76/153)	NS	NS	NS
Ectopic pregnancy rate (%)	0	0	0	–	–	–
Implantation rate (%)	37.1 (13/35)	44.4 (8/18)	48.4 (74/153)	NS	NS	NS
Spontaneous abortion rate (%)	28.6 (4/14)	12.5 (1/8)	22.4 (17/76)	NS	NS	NS
Delivery rate (%)	28.6 (10/35)	38.9 (7/18)	39.2 (60/153)	NS	NS	NS
Live birth rate (%)	28.6 (10/35)	38.9 (7/18)	39.2 (60/153)	NS	NS	NS
Malformation rate (%)	0	0	0	–	–	–
Birth weight (g)	3,357.0 ± 526.6	3,380.7 ± 688.5	3,241.7 ± 574.0	NS	NS	NS
Birth height (cm)	49.6 ± 1.7	49.4 ± 2.9	49.4 ± 2.2	NS	NS	NS

P1, p-value between 0PN and 1PN; P2, p-value between 0PN and 2PN; P3, p-value between 1PN and 2PN; NS, nonsignificant; -, not applicable.

Values are mean ± SD.

a The Mann–Whitney U-test was used for statistical comparison.

During the study period, 12 cycles underwent double blastocyst transfer using 0PN embryos, while 91 cycles had normally developed blastocysts and served as a control group. Only one 1PN cycle is therefore not included. We retrospectively analyzed the cycle of double blastocyst transfer of the same pronuclei state, which refers to transplantation of two 2PN blastocysts or two 0PN blastocysts. Among the patients who received double embryo transfer, no significant differences were found in clinical outcomes between these two groups, including the biochemical pregnancy rate, clinical pregnancy rate, ectopic pregnancy rate, implantation rate, spontaneous abortion rate, delivery rate, live birth rate, and malformation rate (**Table 6**). Similarly, neither birth weight nor birth height was statistically significant in newborn infants (**Table 6**).

**Table 6:** Comparison of double blastocyst transferred cycles resulting in clinical outcomes originating from 0PN and 2PN.

	0PN, 0PN	2PN, 2PN	*P*
Cycle (*n*)	12	91	–
Age (year)	29.8 ± 5.0	31.0 ± 4.4	NS
Times of cumulative transfer per female (*n*)	1.9 ± 1.1	1.9 ± 0.8	NS
Biochemical pregnancy rate (%)	58.3 (7/12)	68.1 (62/91)	NS
Clinical pregnancy rate (%)	58.3 (7/12)	60.4 (55/91)	NS
Ectopic pregnancy rate (%)	0	0	–
Implantation rate (%)	45.8 (11/24)	43.4 (79/182)	NS
Spontaneous abortion rate (%)	28.6 (2/7)	14.5 (8/55)	NS
Delivery rate (%)	50.0 (6/12)	73.6 (67/91)	NS
Live birth rate (%)	50.0 (6/12)	73.6 (67/91)	NS
Malformation rate (%)	0	0	–
Birth weight (g)	3,336.7 ± 789.7	2,921.1 ± 713.0	NS
Birth height (cm)	50.5 ± 1.2	48.0 ± 4.2	NS

P, p-value between 0PN and 2PN, NS, nonsignificant; -, not applicable.

Values are mean ± SD.

## Discussion

Presently, the use of 0PN and 1PN embryos in IVF practice is not recommended by the European Society of Human Reproduction and Embryology (ESHRE) (6).

Our retrospective study showed that embryos with different fertilization statuses (0PN, 1PN, and 2PN) have inconsistent blastocyst development potential. No significant difference was observed when comparing the clinical outcomes obtained from frozen-thawed single or double blastocysts resulting in 0PN or 1PN compared to those achieved with 2PN. The cleavage of 0PN zygotes may be due to normal or abnormal fertilization or parthenogenetic activation (4). It is difficult to determine whether a 0PN embryo originated from one of the above conditions without time-lapse imaging technology. There is a low blastulation rate in cleavage embryos originating from abnormal fertilization and parthenogenetic activation (23, 24). Overall, we found that the frequencies of 0PN and 2PN blastocysts do not differ significantly. This observation is in line with the reported results from (25), showing no significant difference between euploid 0PN and 2PN preimplantation genetic testing for monogenic (PGT-M) blastocysts. However, we found that the development potential of 0PN and 2PN was still different in some specific situations. Although the total blastocyst and good-quality blastocyst rates were consistent, the good-quality day 3 embryo 0PN rate was significantly lower than that of the 2PN. In addition, day 6 0PN embryos had a reduced blastocyst and good-quality blastocyst rate compared to 2PN, suggesting that part of 0PN eliminated aneuploidy during blastocyst formation. It is reported that 0PN blastocysts have similar implantation rates compared to 2PN, but when cleavage-stage embryos were transferred, lower rates were obtained (13). Our findings show that there are no significant differences in clinical outcomes between 0PN and 2PN blastocysts. However, we found that the times of cumulative transfer in women with 0PN embryos were significantly higher than those in women with 2PN embryos, suggesting that the 0PN transfer group in our center suffered from multiple implantation failure times with normal fertilized embryos. Collectively, these observations further strengthen the argument against discarding them in IVF.

The formation of 1PN embryos in conventional IVF cycles can arise from various factors. These include unsynchronized pronuclei formation, the fusion of early pronuclei, or missing the timing for observing bipronuclear development, which may potentially result in the formation of normal diploid embryos. While blastocyst culture is effective at excluding embryos with certain chromosomal abnormalities, it has been observed that 1PN embryos exhibit lower rates of blastocyst formation (7, 14). In our retrospective analysis of 412 1PN embryos, we discovered a limited developmental potential for 1PN embryos. Only 17.0% of 1PN zygotes progressed to the blastocyst stage, and their good-quality blastocyst rates were 7%, which is approximately half of the rate observed in 2PN embryos. Additionally, we found that the rates of blastocyst and good-quality blastocyst formation were consistently lower at each grade level of 1PN embryos on day 3 compared to their 2PN counterparts. However, if the 1PN embryos reached that stage, they can generate pregnancy like 2PN embryos. These findings suggest that a substantial portion of cleavage-stage embryos experienced growth retardation before approaching the blastocyst stage, potentially originating from abnormal fertilization. Blastocyst culture serves as a screening method for identifying embryos with normal chromosomes. In another retrospective cohort study, it was observed that pregnancy rates and live birth rates were lower in single embryo transfer using 1PN embryos compared to 2PN cleavage-stage FET. However, there was no significant difference in outcomes between 1PN and 2PN blastocysts (12). A study by Itoi et al. in 2015 similarly reported that fresh transplantation of 1PN and 2PN blastocysts resulted in similar clinical pregnancy rates (14). Contrary to the findings mentioned above, the levels of IVF 1PN-derived blastocysts were observed to yield lower pregnancy outcomes than expected, even though these embryos can form blastocysts and exhibit no detectable genetic abnormalities (7). Our study demonstrates that the FET of single 1PN blastocyst embryos holds comparable clinical value to that of single 2PN blastocyst embryos. This approach does not increase the risk of miscarriage or ectopic pregnancy, and no congenital malformations were detected in the resulting neonates. It is worth noting that our study only included one case of a live birth from a 1PN double blastocyst FET. Although this particular infant did not exhibit any congenital abnormalities, the limited number of cases highlights the need for further investigation to establish the safety of 1PN-derived double blastocyst transplantation.

This study has several limitations. It is important to note that this research is a single-center retrospective study, and further clarification of our findings can be achieved through a multicenter prospective study. In our reproductive center, pronuclei were routinely observed 19 h after fertilization, which is 1 h later than the standard fertilization time. A retrospective analysis based on time-lapse imaging found that the optimal time to perform fertilization assessment for oocytes cultured in standard incubation is 16.5 h ± 0.5 h postinsemination, and the number of visible pronuclei reduced at 18–18.5 h and continued to fall at 19.5–20 h (26). Kobayashi et al. suggested that using time-lapse imaging, they found that the early fading of PN in growth-accelerated embryos results in misjudged 0PN embryos (27). It is possible that our delayed observation could result in the detection of more disappearing pronuclei. The fate of these embryos is assessed and would have been divided as unfertilized. The embryos judged as 0PN continue to grow to the cleavage stage, and the group of 0PN-derived embryos is overestimated as a result. In spite of this, there are still true 0PN-derived embryos. Therefore, strict observation time after insemination may help reduce missed PN. However, it is worth mentioning that prior studies have reported that observing 1PN embryos 4–6 h after the standard fertilization check resulted in 25% of them being reclassified as 2PN in IVF cycles (5). This underscores the importance of precise timing for fertilization checks, and in the future, we should consider employing time-lapse imaging technology for more accurate observation of pronuclei and embryo evaluation. Furthermore, this study did not include genetic analysis for preimplantation genetic testing for aneuploidies (PGT-A) due to our laboratory not meeting the qualifications for third-generation IVF. PGT should be considered necessary to distinguish aneuploid from 0PN- and 1PN-derived embryos. It is important to note that there were relatively few clinical transplant cycles involving 0PN and 1PN embryos in our research. While we did not find any neonatal malformations, we cannot exclude the possibility that an increased sample size might detect safety defects in offspring. Additionally, this study lacked long-term follow-up regarding the postnatal development of newborns. Congenital malformations at birth are not enough to detect possible anomalies in the children born after the transfer of those embryos. However, a significant proportion of euploid 1PN blastocysts will have maternal uniparental disomy (28). Beyond PGT testing for euploidy, biparental diploidy verification becomes the next direction for exploration, as shown by Soler et al. (28). Chromosomal testing for male and female embryos before IVF cycles is routine. Before 0PN and 1PN embryo transfers, patients must be fully informed. Noninvasive prenatal testing pLUS (NIPT-PLUS), maternal serum screening (MSS), and amniocentesis are all recommended for abnormal fertilization blastocysts in our center. Finally, noninvasive PGT-A (niPGT-A), which is based on sequencing DNA released into the culture medium from both trophectoderm and inner cell mass, may provide a new method for identifying aneuploid in future clinical applications (29).

In summary, our data suggest that 0PN embryos demonstrate a similar ability to form blastocysts during IVF cycles compared to 2PN embryos. Blastocyst cultures, however, do not effectively screen out abnormal 0PN embryos. On the other hand, IVF 1PN embryos, although less likely to develop into blastocysts compared to 2PN embryos, may not carry an additional risk of genetic abnormalities at the blastocyst stage. It remains unclear whether these embryos have lower pregnancy potential. In clinical outcomes, 0PN, 1PN, and 2PN embryos demonstrated similar results, including implantation rates, clinical pregnancy rates, and live birth rates. No congenital malformations were observed. For patients with limited availability of 2PN embryos in routine IVF cycles, 0PN and 1PN embryos can be considered for transplantation after blastocyst culture. We recommend that these embryos be tested prior to transfer, providing infertility with clinically suitable embryo options.

## Conclusions

In conclusion, the results of our present analysis indicate that 1PN embryos exhibit lower developmental potential. There is no significant difference in the 0PN and 2PN developmental potentials. Blastocyst clinical outcomes appear similar among 0PN, 1PN, and 2PN zygotes, but more studies are needed to confirm the safety.

## Data availability statement

The data analyzed in this study is subject to the following licenses/restrictions: The raw data of patients is not publicly available because of patient confidentiality and participant privacy. Requests to access these datasets should be directed to Sheng Yang, ysh20200116@126.com.

## Ethics statement

The studies involving humans were approved by Huzhou Maternity and Child Health Care Hospital. The studies were conducted in accordance with the local legislation and institutional requirements. The participants provided their written informed consent to participate in this study.

## Author contributions

MZ: Writing – original draft, Writing – review & editing. QD: Writing – original draft, Writing – review & editing. YRZ: Writing – review & editing. YL: Writing – review & editing. TW: Writing – review & editing. YPZ: Writing – review & editing. SY: Writing – review & editing.
